# A Novel Codeword Selection Scheme for MIMO-MAC Lower-Bound Maximization

**DOI:** 10.3390/e20080546

**Published:** 2018-07-24

**Authors:** Waleed Shahjehan, Syed Waqar Shah, Jaime Lloret, Ignacio Bosch

**Affiliations:** 1 Department of Electrical Engineering, University of Engineering & Technology, Peshawar P.O. Box 814, Pakistan; 2 Integrated Management Coastal Research Institute, Universitat Politecnica de Valencia, Gandia, 46730 Valencia, Spain; 3 Institute of Telecommunications and Multimedia Applications (iTEAM), Universitat Politècnica de València, Camino Vera n/n, 46022 Valencia, Spain

**Keywords:** Interference Alignment, computational complexity, MAX-SINR, codewords selection, spectral efficiency

## Abstract

Aiming at the limitations of the existing Limited Feedback Interference Alignment algorithms, this paper proposes a direct codeword selection scheme that maximizes the lower-bound of the user rate and reduces the sum rate loss by integrating the Bit Allocation algorithm. The target signal is decoded using the maximum signal to interference plus noise ratio (MAX-SINR) algorithm. Moreover, low complexity and global searching mechanisms are deployed to select the optimized codewords from the generated sets of codewords that approach the ideal precoder. Simulation results show that the proposed algorithm effectively improves the rate lower-bound of the system user as compared with the existing state-of-the-art algorithms.

## 1. Introduction

Multiple-input multiple-output (MIMO) is a technology which can make great enhancements in terms of the overall throughput of the network. How to eliminate the interference of cell edge users in the multi-cell multi-user MIMO downlink has been a research hotspot in recent years. Interference Alignment (IA) can solve the interference problem and increase the achievable rates [1,2]. However, this usually needs to know the local or even global Channel State Information (CSI), and it generally uses the feedback from the receiver. The limited feedback of IA shares the same codebook between the transmitter and receiver, and the receiver quantizes the channel matrix or precoding according to the obtained CSI and sends feedback for the location index of the quantization codeword [3,4]. Because the quantized channel matrix has a larger dimension than the quantized precoding matrix, the quantized precoding scheme has been widely studied [5,6,7,8,9,10,11,12,13,14,15,16,17,18,19,20]. This paper proposes a MIMO Multiple Access Channel (MIMO-MAC) limited feedback IA algorithm that maximizes the rate lower-bound of the system user. It is based on three main steps of operations. First, according to the different channel qualities of each user, bit allocation is performed. After this, selecting the codewords closer to the ideal precoding in the Grassmann codebook space generates an optional set of codewords and adopts the maximum signal to interference plus noise ratio (MAX-SINR) algorithm for decoding. Finally, the codeword combination that can make the user’s rate lower-bound is searched in the set as the optimal quantization precoding. In the proposed algorithm, low-complexity and sub-optimal global search are implemented simultaneously. Simulation results show that compared with other state-of-the-art algorithms, the proposed algorithm effectively improves the user’s sum rate and the lower-bound for the user’s sum rate in the system.

The rest of the paper is organized as follows. Section 2 presents the related work of the paper. Section 3 discusses the system model and analytical derivations. Section 4 explains the proposed algorithm. Section 5 describes the rate loss analysis and bit allocation algorithm of the proposed research work. Section 6 provides the pseudocode and computational complexity of the proposed algorithm. Simulation results and discussions are provided in Section 7 while Section 8 concludes the paper.

## 2. Related Work

Interference Alignment (IA) can effectively solve the interference problem in the interference channel and increase the achievable sum rate of the channel [21,22]. However, it requires global CSI to design the interference suppression matrix, usually using the feedback of the receiver to inform the CSI required by the transmitter [23,24]. In order to save the bandwidth of feedback, the finite feedback of IA shares the same codebook between the transmitter and receiver. The receiver determines the optimal precoding from the global CSI and finds the optimal codeword from the codebook. Then it finds the location index Limited feedback [25,26]. Because the channel matrix dimension is larger than the precoding matrix, the feedback precoding algorithm is better, and the limited feedback of IA achieves greater performance improvement with less feedback and has been widely studied [5,6,7,8,9,10,11,12,13,14,15,16,17,18,19,20].

In the scheme of quantizing channels, the authors in [27] showed that in the L-path frequency selective channel, the degree of freedom (DoF) of K/2 can still be obtained when the receiver has only feedback $K\left(L-1\right)\log_{2}\left(SNR\right)$ bits. In [28], the authors performed Quadratic Reciprocity (QR) decomposition on the joint interference channel to obtain an equivalent unitary matrix and quantized it in the Grassmann codebook, which reduced the performance loss. In the quantized precoding scheme, for the case of a MIMO uplink of two-cells and K users per cell, the authors in [29] align the inter-cell interference (ICI) through inter-base station (BS) interaction CSI joint design precoding. The interference suppression matrix is obtained with the interference in the cell, and finally, the precoding is directly quantized. In [30] and in the literature [29], the ICI and the intra-cell interference cannot be eliminated. In both papers, they first quantified the precoding and redesigned the interference suppression matrix to eliminate the intra-cell interference. In [31], the authors address the problem that ICI cannot be eliminated in [30] and minimize ICI through a direct codeword solution. The authors in [32] extended [30] to several degrees of freedom and improved its performance. The authors in [33] improved the ICI alignment in the direction that is most conducive to receiving rather than that of the traditional randomization direction. On the basis of [33], authors in [34] minimize ICI by jointly selecting quantized codewords, but with high complexity. The authors in [35] prove that using a Grassmann codebook can further improve the system performance. The authors in [36,37] analyze the impact of different decoding algorithms and different system parameters on system performance.

It can be seen that the traditionally limited feedback schemes are based on the criterion of minimum chordal distance [29,30,32] or alignment [31]. It does not consider the overall performance, nor does it consider that the interference suppression matrix may make interference amplification problems. In addition, the joint quantification in [34] is better than the independent quantification performance in [33], but the quality of the signal transmission is not considered; however, the authors in [36,37] only theoretically analyze the performance of limited feedback. Therefore, for the MIMO-MAC case, the codeword can be selected from the perspective of optimizing the overall system performance.

For this reason, this paper proposes a MIMO-MAC limited feedback IA algorithm that maximizes the rate lower-bound of the system user. First, according to the different channel qualities of each user, bit allocation is performed. Secondly, selecting the codewords closer to the ideal precoding in the Grassmann codebook space constitutes an optional set of codewords and adopts the maximum signal to interference plus noise ratio (MAX-SINR) algorithm for decoding. Finally, the codeword combination that can make the user’s maximum rate lower-bound is searched in the set as the optimal quantization precoding. At this time, low-complexity and sub-optimal global search are implemented at the same time. The simulation shows that compared with other algorithms, the proposed algorithm effectively improves the lower-bound of the user’s achievable rate.

## 3. System Model

**Figure 1:**
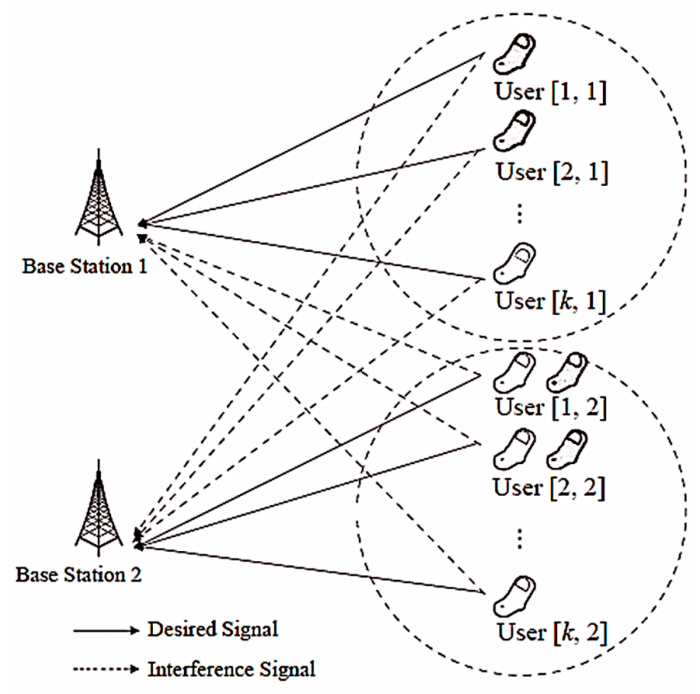
Proposed two-cell multiple-input multiple-output (MIMO)-MAC system model with K users.

This paper considers the MIMO-MAC model consisting of two-cells and K users per cell. The number of transmitting antennas is $N_{t}$ and the number of receiving antennas is $N_{r}$. The system model is shown in Figure 1.

In order to maximize the total DoF, the dimension of the signal space provided by each user should be equal, that is, each user has the same DoF and is assumed to be d. Assume that the channel between each user-BS pair is flat fading and the channel coefficients are independent and identically distributed (i.i.d). The received signal $y_{i}$ of the ith BS for a specific time-frequency can be expressed as:

(1) $$y_{i}=\overset{K}{\underset{l=1}{\sum }}{\left(\frac{d_{0}}{d_{i}^{\left[l,i\right]}}\right)}^{\frac{\gamma }{2}}H_{i}^{\left[l,i\right]}V^{\left[l,i\right]}s^{\left[l,i\right]}+\overset{K}{\underset{m=1,j\neq 1}{\sum }}{\left(\frac{d_{0}}{d_{i}^{\left[m,j\right]}}\right)}^{\frac{\gamma }{2}}H_{i}^{\left[m,j\right]}V^{\left[m,i\right]}s^{\left[m,i\right]}+n_{i}$$

where $d_{0}$ is the reference distance, $d_{i}^{\left[l,i\right]}$ and $d_{i}^{\left[m,j\right]}$ represent the propagation distances from user [l,i] and the d [m,j] to ith BS, respectively. γ is the path loss exponent. $H_{i}^{\left[l,i\right]}\in {\mathbb{C}}^{N_{r}\times N_{t}}$ and $H_{i}^{\left[m,j\right]}\in {\mathbb{C}}^{N_{r}\times N_{t}}$ represent the channel matrix from the user [l,i] and [m,j] to the ith BS, respectively, which follows the complex Gaussian distribution with zero mean and unit variance. $V^{\left[l,i\right]}\in {\mathbb{C}}^{N_{t}\times d}$ and $V^{\left[m,i\right]}\in {\mathbb{C}}^{N_{t}\times d}$ are the precoding matrices for users [l,i] and [m,j] corresponding to ith and jth BSs, respectively, which satisfy ${\left(V^{\left[l,i\right]}\right)}^{H}V^{\left[l,i\right]}=I_{d}$ and ${\left(V^{\left[m,j\right]}\right)}^{H}V^{\left[m,j\right]}=I_{d}$. $s^{\left[l,i\right]}\in {\mathbb{C}}^{d\times 1}$ and $s^{\left[m,i\right]}\in {\mathbb{C}}^{d\times 1}$ are the uplink data vector signals for users [l,i] and [m,j] which satisfy the power constraints $E\left[\|s^{\left[l,i\right]}{\|}^{2}\right]=P^{\left[l,i\right]}$ and $E\left[\|s^{\left[m,j\right]}{\|}^{2}\right]=P^{\left[m,j\right]}$, respectively. $P^{\left[l,i\right]}$ and $P^{\left[m,j\right]}$ represent the transmit power for the users [l,i] and [m,j], respectively. $n_{i}\in {\mathbb{C}}^{N_{r}\times 1}$ is the additive white Gaussian noise with zero mean and a variance of $\delta^{2}$, that is, $E\left[n_{i}n_{i}^{H}\right]=\delta^{2}I_{N_{r}}$.

The BS uses the receiver filter $U^{\left[k,i\right]}$ for processing. At this time, the received signal $y_{\left[k,i\right]}$ for the user [k,i] is:

(2) $$\begin{array}{c} y_{\left[k,i\right]}=U^{\left[k,i\right]H}{\left(\frac{d_{0}}{d_{i}^{\left[k,i\right]}}\right)}^{\frac{\gamma }{2}}H_{i}^{\left[k,i\right]}V^{\left[k,i\right]}s^{\left[k,i\right]}+U^{\left[k,i\right]H}\overset{K}{\underset{l=1,l\neq k}{\sum }}{\left(\frac{d_{0}}{d_{i}^{\left[l,i\right]}}\right)}^{\frac{\gamma }{2}}H_{i}^{\left[l,i\right]}V^{\left[l,i\right]}s^{\left[l,i\right]}\\ +U^{\left[k,i\right]H}\overset{K}{\underset{m=1,j\neq i}{\sum }}{\left(\frac{d_{0}}{d_{i}^{\left[m,j\right]}}\right)}^{\frac{\gamma }{2}}H_{i}^{\left[m,j\right]}V^{\left[m,j\right]}s^{\left[m,j\right]}+U^{\left[k,i\right]H}n_{i} \end{array}$$

## 4. New Limited Feedback Interference Alignment Algorithm

### 4.1. Precoding Matrix and Codeword Selection Scheme

From the perspective of IA, the aligned ICI channels, from users in the ith cell to the jth BS, span the intersection subspace $H_{j}^{\mathrm{ICI}}$, which can be expressed as:

(3) $$\begin{array}{ll} \mathrm{span}\left(H_{j}^{\mathrm{ICI}}\right) & =\mathrm{span}\left(H_{i}^{\left[l,i\right]}V^{\left[l,i\right]}\right)\\ & =\ldots =\mathrm{span}\left(H_{i}^{\left[K,i\right]}V^{\left[K,i\right]}\right) \end{array}$$

where $H_{i}^{\left[l,i\right]}$ and $V^{\left[l,i\right]}$ represent the channel matrix for the user [l,i] to the ith BS, respectively. The pre-encoded and aligned interference term $H_{j}^{\mathrm{ICI}}$ that satisfies Equation (3) can be obtained by Equation (4):

(4) $$\left[\begin{array}{c} I_{N_{r}}\\ I_{N_{r}}\\ \vdots \\ I_{N_{r}} \end{array}\;\begin{array}{c} -H_{j}^{\left[1,i\right]}\\ 0\\ \vdots \\ 0 \end{array}\;\begin{array}{c} 0\\ -H_{j}^{\left[2,i\right]}\\ \vdots \\ 0 \end{array}\;\begin{array}{c} \ldots \\ \ldots \\ \ddots \\ \ldots \end{array}\;\begin{array}{c} 0\\ 0\\ \vdots \\ -H_{j}^{\left[K,i\right]} \end{array}\right]\left[\begin{array}{c} H_{j}^{\mathrm{ICI}}\\ {\tilde{V}}^{\left[1,i\right]}\\ {\tilde{V}}^{\left[2,i\right]}\\ \vdots \\ {\tilde{V}}^{\left[K,i\right]} \end{array}\right]=0$$

It can be known from [38] that when sending multiple data streams, Schmidt orthogonalization is performed on the pre-coded column vectors, which can further increase the user rate. Therefore, we consider using Schmidt orthogonalization to handle precoding, which can be expressed as:

(5) $$V^{\left[K,i\right]}=\mathrm{orth}\left({\tilde{V}}^{\left[k,i\right]}\right)$$

where “orth” denotes Schmidt orthogonalization. According to the theory of matrix, we can know that:

(6) $$\mathrm{span}\left(H_{j}^{\left[k,i\right]}V^{\left[k,i\right]}\right)=\mathrm{span}\left(H_{j}^{\left[k,i\right]}{\tilde{V}}^{\left[k,i\right]}\right)$$

where $V^{\left[k,i\right]}$ is the column generation space for ${\tilde{V}}^{\left[k,i\right]}$, $H_{j}^{\left[k,i\right]}{\tilde{V}}^{\left[k,i\right]}$, and $H_{j}^{\left[k,i\right]}V^{\left[k,i\right]}$, and they span the same space. So, using $V^{\left[k,i\right]}$ as the precoding does not affect the IA constraint of Equation (3).

Under ideal CSI, precoding can be completely aligned to ICI according to Equations (4) and (5). Zero-Forcing (ZF) processing at the receiver can extract useful signals. With limited feedback, the achievable rate for the user [k,i] can be expressed as:

(7) $$R^{\left[k,i\right]}=\overset{d}{\underset{q=1}{\sum }}\log_{2}\left(1+\frac{P_{i}^{\left[k,i\right]}\left|{\hat{u}}_{q}^{\left[k,i\right]H}H_{i}^{\left[k,i\right]}{\hat{v}}_{q}^{\left[k,i\right]}\right|}{d\;\|{\hat{u}}_{q}^{\left[k,i\right]}{\|}^{2}\delta^{2}+I_{q}^{\left[k,i\right]}}\right)$$

where

(8) $$\begin{array}{c} I_{q}^{\left[k,i\right]}=\underset{\mathrm{ISI}}{\underbrace{\overset{d}{\underset{n=1,\;n\neq q}{\sum }}\frac{P_{i}^{\left[k,i\right]}}{d}\;{\left|{\hat{u}}_{q}^{\left[k,i\right]H}H_{i}^{\left[k,i\right]}{\hat{v}}_{n}^{\left[k,i\right]}\right|}^{2}}}+\underset{\mathrm{IUI}}{\underbrace{\overset{K}{\underset{j=1,\;j\neq k}{\sum }}\frac{P_{i}^{\left[k,i\right]}}{d}\;{\left|{\hat{u}}_{q}^{\left[k,i\right]H}H_{i}^{\left[j,i\right]}{\hat{v}}^{\left[j,i\right]}\right|}^{2}}}\\ +\underset{\mathrm{ICI}}{\underbrace{\overset{2}{\underset{\begin{array}{c} w=1,\;\\ w\neq i \end{array}}{\sum }}\overset{K}{\underset{m=1}{\sum }}\frac{P_{i}^{\left[m,w\right]}}{d}{\left|{\hat{u}}_{q}^{\left[k,i\right]H}H_{i}^{\left[m,w\right]}{\hat{v}}^{\left[m,w\right]}\right|}^{2}}} \end{array}$$

In Equation (8), the first, second, and third terms represent the interference leakage caused by Inter-symbol Interference (ISI), Inter-user Interference (IUI), and Inter-cell Interference (ICI), respectively. The ${\hat{u}}_{q}^{\left[k,i\right]}$ and ${\hat{v}}_{q}^{\left[k,i\right]}$ represent the qth column of the interference suppression matrix ${\hat{u}}^{\left[k,i\right]}$ and the quantization precoding ${\hat{v}}^{\left[k,i\right]}$ matrix, respectively. $P_{i}^{\left[k,i\right]}={\left(\frac{d_{0}}{d_{i}^{\left[k,i\right]}}\right)}^{\gamma }P^{\left[k,i\right]}$ and $P_{i}^{\left[m,j\right]}={\left(\frac{d_{0}}{d_{i}^{\left[m,j\right]}}\right)}^{\gamma }P^{\left[m,j\right]}$ denote the signal power for the user [k,i] and the user [m,j], respectively, when the signal propagates to the ith BS. 

According to the minimum chordal distance criterion, which guarantees that the angle between the precoding and the ideal precoding is the minimum, but after processing by the receiving filter matrix, the interference may be amplified, and, therefore, Equation (8) cannot be guaranteed to be the minimum [12]. In addition, the use of independent quantitative precoding will also cause some users to receive more severe interference. In summary, it can be seen that the limited feedback needs to consider two factors comprehensively. That is, smaller chordal distance and less interference can improve the overall performance. For this reason, this paper establishes a feasible region and finds the optimal codeword combination through a low complexity search within a small distance from the ideal precoding string. 

The specific implementation is as follows. Calculate the chordal distance between the ideal precoding and all codewords in the codebook which can be expressed as:

(9) $$\phi \left(c_{x},V^{\left[k,i\right]}\right)=\frac{1}{\sqrt{2}}\|c_{x}c_{x}^{H}-V^{\left[k,i\right]}V^{\left[k,i\right]H}{\|}_{F}$$

where $c_{x}\in C$ and$\;C=\left\{c_{1},c_{2},\;\ldots ,\;c_{2^{B^{\left[k,i\right]}}}\right\}$. It is worth noting that each $c_{x}$ is a semi-unitary matrix, and $B^{\left[k,i\right]}$ is the number of bits fed back by the user [k,i]. With limited feedback, the optimal region of the precoding matrix for user [k,i] is:

(10) $${\bar{C}}_{\mathrm{opt}}^{\left[k,i\right]}=\varphi_{g}\left(C,\;V^{\left[k,i\right]}\right)$$

where $\varphi_{g}$ denotes finding g codewords with minimum distance from $V^{\left[k,i\right]}$ in codebook C.

### 4.2. Decoding Algorithm Improvement

In the case of quantitative precoding determination, the ZF algorithm [32] is not optimal for decoding. Therefore, this paper considers the decoding of the MAX-SINR algorithm. For the *q*th data stream for the user [k,i], the SINR is expressed as:

(11) $$\mathrm{SINR}=\frac{\mathrm{trace}\left({\hat{u}}_{q}^{\left[k,i\right]H}\;W^{\left[k,i\right]}{\hat{u}}_{q}^{\left[k,i\right]}\right)}{\mathrm{trace}\left({\hat{u}}_{q}^{\left[k,i\right]H}\;F^{\left[k,i\right]}{\hat{u}}_{q}^{\left[k,i\right]}\right)}$$

where $W^{\left[k,i\right]}$ and $F^{\left[k,i\right]}$ matrices are calculated as follows:

(12) $$W^{\left[k,i\right]}=\frac{P_{i}^{\left[k,i\right]}}{d}H^{\left[k,i\right]}{\hat{v}}_{q}^{\left[k,i\right]}{\hat{v}}_{q}^{\left[k,i\right]H}H^{\left[k,i\right]H}$$

(13) $$F^{\left[k,i\right]}=\overset{d}{\underset{n=1,\;n\neq q}{\sum }}\frac{P_{i}^{\left[k,i\right]}}{d}H_{i}^{\left[k,i\right]}{\hat{v}}_{n}^{\left[k,i\right]}{\hat{v}}_{n}^{\left[k,i\right]H}H_{i}^{\left[k,i\right]H}+\overset{K}{\underset{j=1,\;j\neq k}{\sum }}\frac{P_{i}^{\left[j,i\right]}}{d}H_{i}^{\left[j,i\right]}{\hat{v}}^{\left[j,i\right]}{\hat{v}}^{\left[j,i\right]H}H_{i}^{\left[j,i\right]H}\;+\overset{2}{\underset{\begin{array}{c} w=1\\ w\neq i \end{array}}{\sum }}\overset{K}{\underset{m=1}{\sum }}\frac{P_{i}^{\left[m,w\right]}}{d}H_{i}^{\left[m,w\right]}{\hat{v}}^{\left[m,w\right]}{\hat{v}}^{\left[m,w\right]H}H_{i}^{\left[m,w\right]H}+\delta^{2}I_{N_{r}}$$

Since $W^{\left[k,i\right]}$ and $F^{\left[k,i\right]}$ are Hermita matrices and are positive definite. According to the definition of the generalized feature space, the existence of the $N_{r}\times N_{r}$ dimensional matrix $T^{\left[k,i\right]}$ makes the following conditions satisfied:

(14) $$T^{\left[k,i\right]H}W^{\left[k,i\right]}T^{\left[k,i\right]}=\Lambda_{\left[k,i\right]}$$

(15) $$T^{\left[k,i\right]H}F^{\left[k,i\right]}T^{\left[k,i\right]}=I_{N_{r}}$$

where the column vector of the matrix $T^{\left[k,i\right]}$ is the generalized eigenvector of the matrix $\left\{W^{\left[k,i\right]},F^{\left[k,i\right]}\right\}$. The main diagonal elements of the diagonal array $\Lambda_{\left[k,i\right]}$ are all non-negative real numbers and are arranged in descending order with $\mathrm{rank}\left(W^{\left[k,ithe\right]}\right)=1$ and $\mathrm{rank}\left(\Lambda_{\left[k,i\right]}\right)=1$. For this, just take ${\hat{u}}_{q}^{\left[k,i\right]}$ as the first column of $T^{\left[k,i\right]}$. So, the unit vector ${\hat{u}}_{q}^{\left[k,i\right]}$ maximizing the SINR is expressed as:

(16) $${\hat{u}}_{q}^{\left[k,i\right]}=v_{\max\;}\left({\left(F^{\left[k,i\right]}\right)}^{-1}W^{\left[k,i\right]}\right)$$

where $v_{\max\;}\left(A\right)$ represents the unit eigenvector corresponding to the maximum eigenvalue of matrix A.

## 5. Rate Loss Analysis and Bit Allocation Algorithm

### 5.1. Rate Loss Analysis

In limited feedback, it can be known from [23] that the performance of decoding using Equation (16) will be better than that of [32], and the user’s rate loss will be less than the literature [32]. So, there is a condition:

(17) $$I_{q}^{\left[k,i\right]}\leq \Gamma_{q,\;\mathrm{IC}}^{\left[k,i\right]}$$

where $I_{q}^{\left[k,i\right]}$ and $\Gamma_{q,\;\mathrm{IC}}^{\left[k,i\right]}$ indicate the interference leakage of the *q*th data stream of user [k,i] when using the proposed algorithm and the literature [32], respectively. The average power of the interference leakage under limited feedback can be calculated as:

(18) $$E\left[I_{q}^{\left[k,i\right]}\right]\leq E\left[\left(\overset{K}{\underset{l=1,\;j\neq i}{\sum }}\frac{P_{i}^{\left[l,j\right]}}{d}\;{\tilde{\Delta }}^{\left[l,j\right]}\right)\right]$$

where ${\tilde{\Delta }}^{\left[l,j\right]}=\|{\hat{v}}^{\left[l,j\right]}{\hat{v}}^{\left[l,j\right]H}-V^{\left[l,j\right]}V^{\left[l,j\right]H}{\|}_{F}^{2}$. When the codebook is generated in a sphere-packing procedure, the upper bound on the maximum value of the quantization error is given as [38]:

(19) $$\Delta_{\max}^{\left[l,j\right]}=\max_{V^{\left[l,j\right]}\in G_{N_{t,d}}}\;{\tilde{\Delta }}^{\left[l,j\right]}\leq \frac{8}{{\left(c2^{B^{\left[l,j\right]}}\right)}^{\frac{2}{N_{G}}}}$$

where the constant c is the coefficient of the ball volume in the Grassmannian manifold and $N_{G}=2d\left(N_{t}-d\right)$ [38]. Putting Equation (19) into (18), we have the upper-bound of the average power of the overall interference leakage as:

(20) $$E\left[I_{q}^{\left[k,i\right]}\right]\leq \overset{K}{\underset{l=1,\;j\neq i}{\sum }}\frac{8P_{i}^{\left[l,j\right]}}{d{\left(c2^{B^{\left[l,j\right]}}\right)}^{\frac{2}{N_{G}}}}$$

### 5.2. Bit Allocation Algorithm

When the total number of feedback bits in the system is $B_{T}=\sum_{i=1}^{2}\sum_{k=1}^{K}B^{\left[k,i\right]}$ and is fixed, the interference leakage can be reduced, and sum rate can be improved by minimizing the expected quantization error due to ICI through an adaptive bit allocation algorithm. Combined with Equation (20), the optimization problem can be described as follows:

(21) $$\min\overset{2}{\underset{i=1}{\sum }}\overset{K}{\underset{k=1}{\sum }}Q_{r}^{\left[k,i\right]}2^{\frac{-2B^{\left[k,i\right]}}{N_{G}}}\;s.t.\;\overset{2}{\underset{i=1}{\sum }}\overset{K}{\underset{k=1}{\sum }}B^{\left[k,i\right]}\leq B_{T}$$

where $Q_{r}^{\left[k,i\right]}=\frac{8P_{i}^{\left[l,j\right]}}{\left(dc^{\frac{2}{N_{G}}}\right)}$.

Strictly solving Equation (21) will be very complicated since it is a non-linear integer problem and requires very high computational complexity as the total number of feedback bits increases. Since the objective function is log-convex-optimized, we can use a convex optimization technique to obtain the closed-form solution of Equation (21). The Lagrangian function expression of Equation (21) is as follows:

(22) $$L\left(B^{\left[k,i\right]},\;\lambda \right)=\overset{2}{\underset{i=1}{\sum }}\overset{K}{\underset{k=1}{\sum }}Q_{r}^{\left[k,i\right]}2^{\frac{-2B^{\left[k,i\right]}}{N_{G}}}+\lambda \left(\overset{2}{\underset{i=1}{\sum }}\overset{K}{\underset{k=1}{\sum }}B^{\left[k,i\right]}-B_{T}\right)$$

From Equation (22), we find the first order optimality Karush-Kuhun-Tucker (KKT) conditions as:

(23) $$\frac{\partial L\left(B^{\left[k,i\right]},\lambda \right)}{\partial B^{\left[k,i\right]}}=-\frac{2\ln\left(2\right)Q_{r}^{\left[k,i\right]}}{N_{G}}2^{\frac{-2B^{\left[k,i\right]}}{N_{G}}}+\lambda =0$$

(24) $$\frac{\partial L\left(B^{\left[k,i\right]},\lambda \right)}{\partial \lambda }=\overset{2}{\underset{i=1}{\sum }}\overset{K}{\underset{k=1}{\sum }}B^{\left[k,i\right]}-B_{T}=0$$

By solving Equations (23) and (24), we get the suboptimal solution:

(25) $$B^{\left[k,i\right]\;*}=\min\left\{B_{T},\;\lfloor \frac{B_{T}}{2K}+\frac{N_{G}}{2}\log_{2}\left(\frac{Q_{r}^{\left[k,i\right]}}{{\left(\prod_{t=1}^{2}\prod_{w=1}^{K}Q_{r}^{\left[w,t\right]}\right)}^{\frac{1}{2K}}}\right)\rfloor^{+}\right\}$$

where $\lfloor \;x\;\rfloor^{+}=\max\left(0,\;\lfloor \;x\;\rfloor \right)$ and ⌊ x ⌋ means to find the largest integer not greater than x.

## 6. Algorithm Summary and Theoretical Performance Analysis

### 6.1. Algorithm Summary

Through the above analysis, the limited feedback IA algorithm (Algorithm 1) is summarized as follows:

**Algorithm 1** Limited Feedback IA**Step 1:** Determine the bit allocation using Equations (25) according to the user’s channel quality. **Step 2:** Obtain the ideal precoding according to Equations (4) and (5), according to the ideal CSI. **Step 3:** Generate the precoding feasible region according to Equations (9) and (10), according to the ideal precoding obtained in Step 2. **Step 4:** Select one code word for each user in the feasible field and generate a user quantized precoding combination. **Step 5:** Perform decoding according to Equation (16), according to the obtained quantized precoding combination. **Step 6:** Calculate the channel capacity of all users and let the smallest element in the channel capacity set be γ. **Step 7:** Repeat steps 4 and 5 until a codeword combination that maximizes γ is found in the precoding feasible region, and the corresponding codeword combination is used as the optimal quantization precoding combination. **Step 8:** Feedback the location index of the best-quantized precoding in the codebook to the transmitter.

### 6.2. User Rate Lower-Bound Analysis

In limited feedback, the overall performance can only be improved by only considering a small chordal distance and less interference. For this reason, this paper establishes a smaller feasible region around the ideal precoding and finds the optimal combination of codewords by simply searching. The idea of IA is to divide the interference and signal into independent subspaces. The design of the interference suppression matrix is considered from the perspective of balancing the interference between each other using a joint strategy [3]. In the MIMO-MAC model, from the perspective of space, if the ICI can be completely aligned, then only a simple ZF processing needs to be performed at the receiver. On the other hand, the ICI processing can also be analyzed to be better or poorer. This paper maximizes the lower limit of user rates, assuming that cell 1 is used. When the lower limit of the user 1 rate is increased, then it can be seen that the interference of cell 2 to cell 1 is reduced, and the user rate in cell 1 is improved. The quantitative precoding for the user in fixed cell 2 finds cell 1 by further searching the codeword. The reduced codewords for cell 2 further enhance the user’s rate in cell 2. Through such overlapping searches, the optimal codeword combination can be obtained in the feasible region. For this reason, such searches not only increase the lower limit of the system user rate but also improve the spectral efficiency (SE). 

### 6.3. Complexity Analysis

According to the deduction, the user rate loss is:

(26) $$\begin{array}{ll} \Delta R^{\left[k,i\right]} & =R_{\mathrm{PFB}}^{\left[k,i\right]}-R_{\mathrm{LFB}}^{\left[k,i\right]}\\ & =E\left[\overset{d}{\underset{q=1}{\sum }}\log_{2}\left(1+\frac{P_{i}^{\left[k,i\right]}}{d}\;\frac{{\left|u_{q}^{\left[k,i\right]H}H_{i}^{\left[k,i\right]}v_{q}^{\left[k,i\right]}\right|}^{2}}{\delta^{2}}\right)\right]\\ & -E[\overset{d}{\underset{q=1}{\sum }}\log_{2}\left(1+\frac{P_{i}^{\left[k,i\right]}}{d}\;\frac{{\left|{\hat{u}}_{q}^{\left[k,i\right]H}H_{i}^{\left[k,i\right]}{\hat{v}}_{q}^{\left[k,i\right]}\right|}^{2}}{\delta^{2}+I_{q}^{\left[k,i\right]}}\right)]\\ & \leq E[\overset{d}{\underset{q=1}{\sum }}\log_{2}\left(\delta^{2}+\frac{P_{i}^{\left[k,i\right]}}{d}\;{\left|u_{q}^{\left[k,i\right]H}H_{i}^{\left[k,i\right]}v_{q}^{\left[k,i\right]}\right|}^{2}\right)]\\ & -E[\overset{d}{\underset{q=1}{\sum }}\log_{2}\left(\delta^{2}+\frac{P^{\left[k,i\right]}}{d}\;{\left|{\hat{u}}_{q}^{\left[k,i\right]H}H^{\left[k,i\right]}{\hat{v}}_{q}^{\left[k,i\right]}\right|}^{2}\right)]\\ & +E[\overset{d}{\underset{q=1}{\sum }}\log_{2}\left(\frac{\delta^{2}+I_{q}^{\left[k,i\right]}}{\delta^{2}}\right)]\\ & \leq \overset{d}{\underset{q=1}{\sum }}\log_{2}\left(\frac{\delta^{2}+E\left(I_{q}^{\left[k,i\right]}\right)}{\delta^{2}}\right) \end{array}$$

The first inequality is determined by the $\log_{2}\left(\cdot \right)$ increasing function and $I_{q}^{\left[k,i\right]}\geq 0$. The second inequality is because $u_{q}^{\left[k,i\right]H}$ and ${\hat{u}}_{q}^{\left[k,i\right]H}$ are evenly distributed in ${\mathbb{C}}^{N_{r}\times d}$ space. $v_{q}^{\left[k,i\right]}$ and ${\hat{v}}_{q}^{\left[k,i\right]}$ are also uniformly distributed in ${\mathbb{C}}^{N_{t}\times d}$ space and are obtained by Jensen inequality. Putting Equation (20) into Equation (26) we get:

(27) $$\Delta R^{\left[k,i\right]}\leq \overset{d}{\underset{q=1}{\sum }}\log_{2}\left(1+\overset{K}{\underset{l=1,\;j\neq i}{\sum }}\frac{8P_{i}^{\left[l,j\right]}}{\delta^{2}d{\left(c2^{B^{\left[l,j\right]}}\right)}^{\frac{2}{N_{G}}}}\right)$$

From Equation (27), we can see that $\frac{P_{i}^{\left[l,j\right]}}{d}$ is linearly proportional to ${\left(c2^{B^{\left[l,j\right]}}\right)}^{\frac{2}{N_{G}}}$ in order to guarantee the same DoF as the ideal CSI. Therefore, there is:

(28) $$\frac{P_{i}^{\left[l,j\right]}}{d}=\lambda {\left(c2^{B^{\left[l,j\right]}}\right)}^{\frac{2}{N_{G}}}$$

where λ(λ>0) is a scale factor. Because the DoF is defined as $P_{i}^{\left[l,j\right]}\to +\infty$, in order to achieve the DoF, it is known from Equation (28). The number of feedback bits can be expressed as:

(29) $$B^{\left[k,i\right]}=d\left(N_{t}-d\right)\log_{2}\left(P_{i}^{\left[l,j\right]}\right)$$

In order to obtain the optimal precoding matrix, [9,10,12] require the number of searches to be $2^{b+1}K,$ [11] requires $2^{bK+1}$, and [14] requires $2^{Kb+1}$. In order to obtain the optimal precoding matrix, the proposed algorithm requires $2^{gK}+2^{b+1}K$. It can be seen that the complexity of the proposed algorithm is higher than that of [9,10,12] but lower than [11,14].

### 6.4. Number of Required Antennas

For the 2-user MIMO-MAC channel, in each cell of 2 cells for the total DoF to be 4, the transmitter in [13] needs 3 antennas and the receiver needs at least 3 antennas. In [9,10,11,12,14], by aligning the ICIs together, only 2 transmitter antennas and 3 receiver antennas are required, and a DoF of 4 can be obtained. From Equation (4), we can determine that if we have a solution (4) and each user gets a DoF of d, then there must be:

(30) $$\left(KN_{t}+N_{r}\right)-\left(KN_{r}\right)\geq d$$

## 7. Simulation Results and Analysis

We deployed MATLAB for performing the analysis and experimentation. Consider the system configuration of ${\left[K,d,\;\left(N_{r}\times N_{t}\right)\right]}^{2}$. That is, two cells, K users per cell, a MIMO-MAC model with a DoF of d for each user, and the number of BS antennas is $N_{t}$. The number of subscriber antennas is $N_{r}$. All channels are flat Rayleigh fading, and their elements satisfy a cyclic symmetric complex Gaussian distribution with a zero mean and unit variance. The channel noise is an Additive White Gaussian Noise with a zero mean and unit variance. Finally, the proposed algorithm is compared with the algorithms of [9,12,14]. All the simulations take 10,000 channel averages.

### 7.1. Average Spectral Efficiency under Ideal CSI

**Figure 2:**
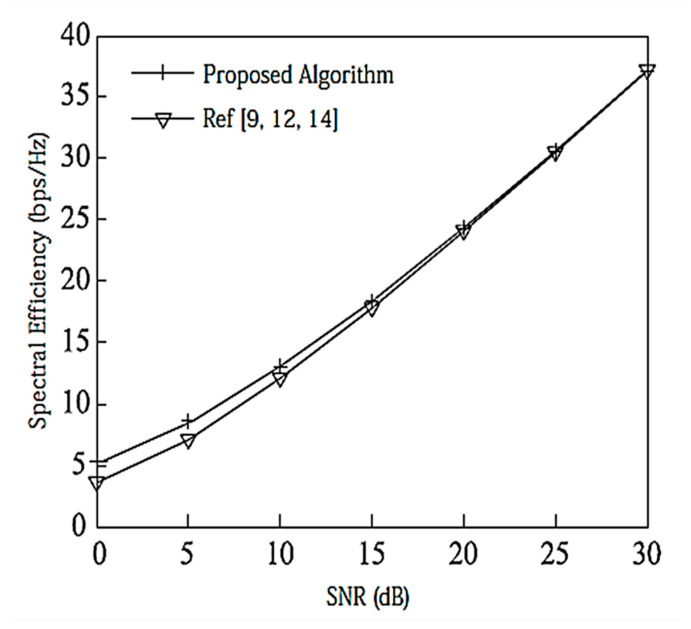
Spectral efficiency analysis of the ${\left[2,1,\;\left(3\times 2\right)\right]}^{2}$ system configuration.

Figure 2 compares the SE of the proposed algorithm with other state-of-the-art algorithms. We assume that there is no power loss when the signal propagates to the BS. Under the configuration of ${\left[2,1,\;\left(3\times 2\right)\right]}^{2}$, since there is no interference between data streams, the performance of [9,12,14] is the same and the MAX Signal-to-Interference plus Noise Ratio (MAX-SNR) is used in this paper. The proposed algorithm has better performance than the existing algorithms.

**Figure 3:**
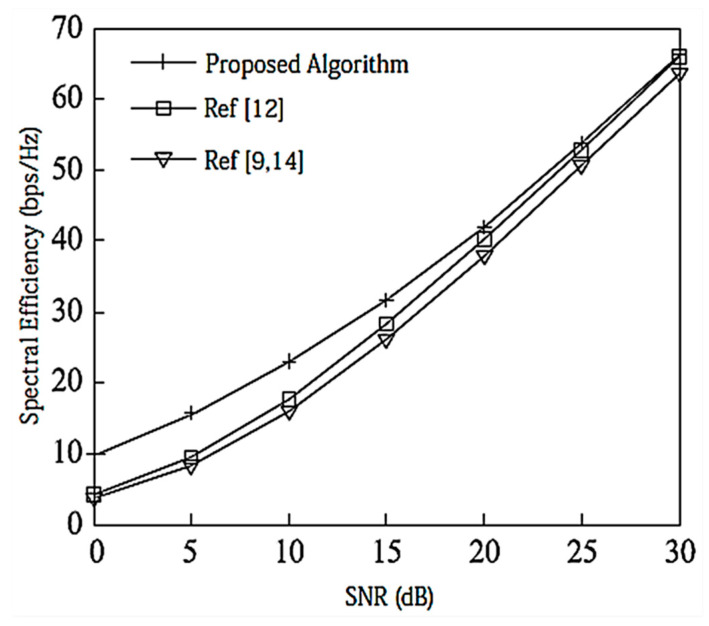
Spectral efficiency analysis of the ${\left[2,2,\;\left(6\times 4\right)\right]}^{2}$ system configuration.

Under the configuration of ${\left[2,2,\;\left(6\times 4\right)\right]}^{2}$, as shown in Figure 3, the DoF for each user is 2, and [12] considers the correlation between user data streams when designing the precoding matrix, and its performance is better than that of [9,14]. In this paper, the precoding matrix is designed to reduce the interference between data streams by using a Schmidt positive-cost reduction, and the decoding is performed using the MAX-SINR algorithm. Therefore, the proposed IA algorithm has better SE than [12].

### 7.2. Average Spectral Efficiency under Limited Feedback CSI

**Figure 4:**
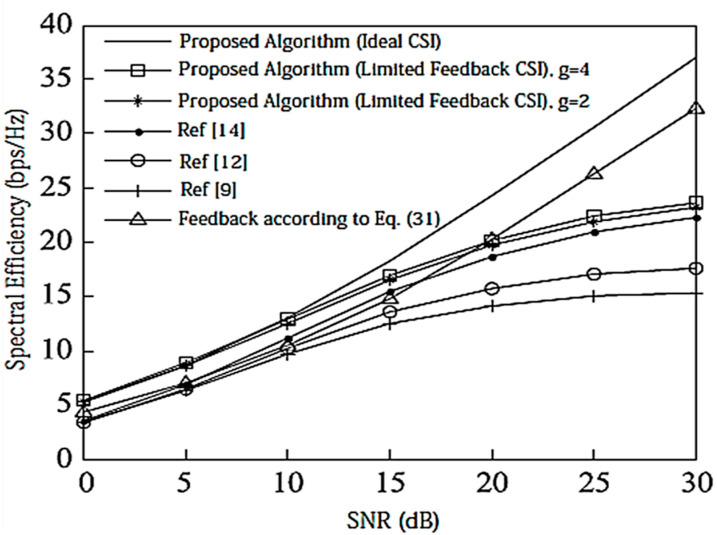
Spectral efficiency analysis of the ${\left[2,1,\;\left(3\times 2\right)\right]}^{2}$ system configuration when bit = 6. CSI = Channel State Information.

**Figure 5:**
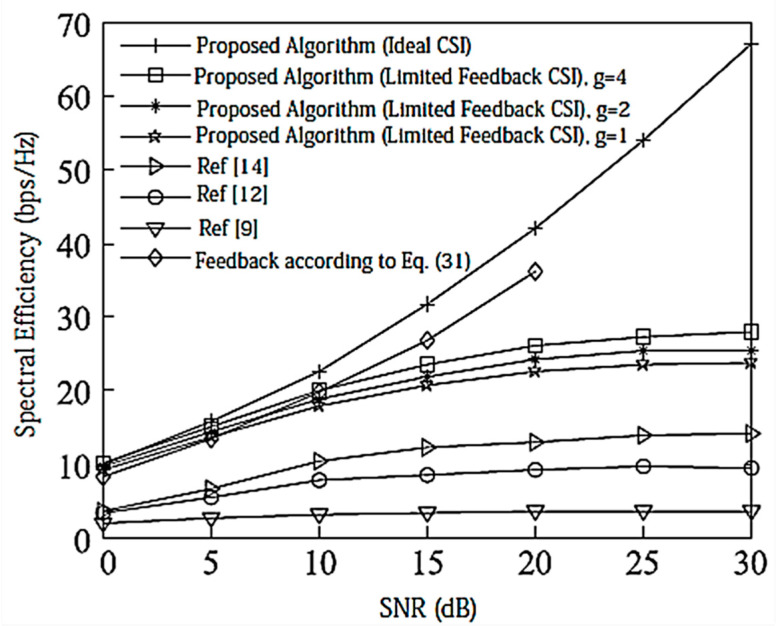
Spectral efficiency analysis of the ${\left[2,2,\;\left(6\times 4\right)\right]}^{2}$ system configuration when bit = 8.

Assume here that there is no power loss when the signal propagates to the BS. When the system is configured as ${\left[2,1,\;\left(3\times 2\right)\right]}^{2}$ and ${\left[2,2,\;\left(6\times 4\right)\right]}^{2}$, the algorithm passes through the receiver SINR, and searches for the optimal codeword in the codebook domain close to the ideal precoding, which can further improve the system performance. In the multi-DoF transmission, Schmidt orthogonalization is performed on precoding, which further weakens the interference between user data streams. From Figure 4 and Figure 5, it can be seen that [14] adopts a joint quantification strategy and its performance is better than [9,12], but the joint selection strategy of the proposed IA algorithm using the maximum SINR is relative to that of [14]. The joint strategy of the proposed IA algorithm to minimize the interference leakage is better than that of [9,12,14]. In addition, as can also be seen from the Figure 4 and Figure 5, according to Equation (31), the number of feedback bits can indeed guarantee the DoF of the system.

**Figure 6:**
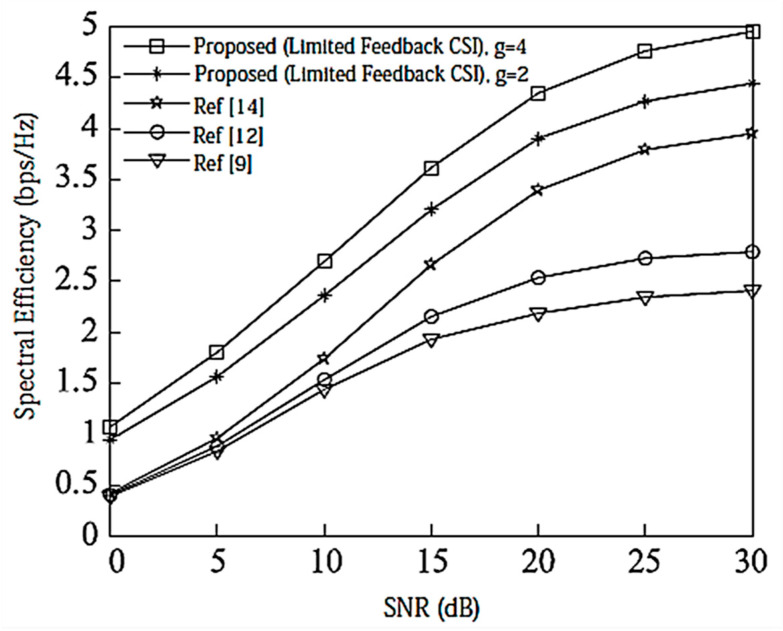
The average lower limit of the system user rate at ${\left[2,1,\;\left(3\times 2\right)\right]}^{2}$ and bit = 6.

**Figure 7:**
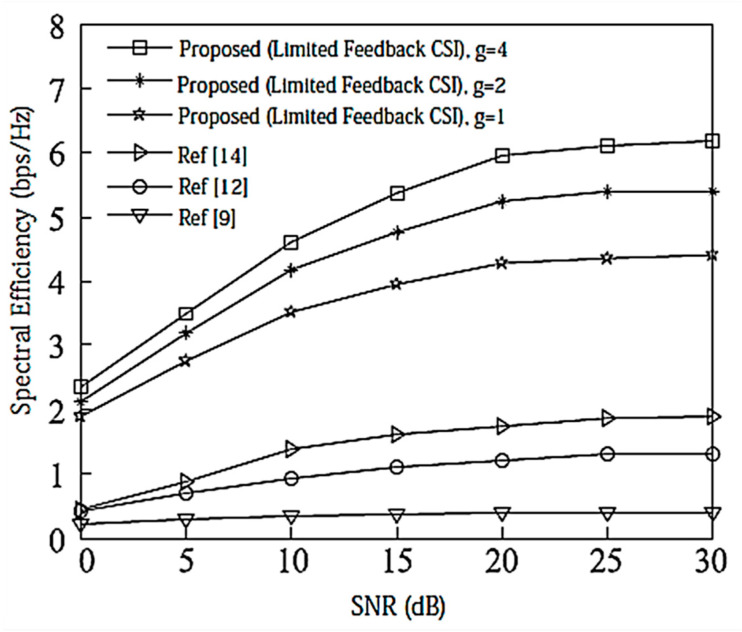
The average lower limit of the system user rate at ${\left[2,2,\;\left(6\times 4\right)\right]}^{2}$ and bit = 8.

According to the analysis in Section 6, the proposed IA algorithm cannot only increase the sum rate of the system, but it also increases the lower limit of the system user rate. For this reason, Figure 6 and Figure 7 compare the lower limit of the system user rate when using different algorithms. From these two figures, it can be seen that the proposed algorithm effectively improves the overall system performance, and increasing the search range appropriately can further increase the lower limit bound of the system user rate.

**Figure 8:**
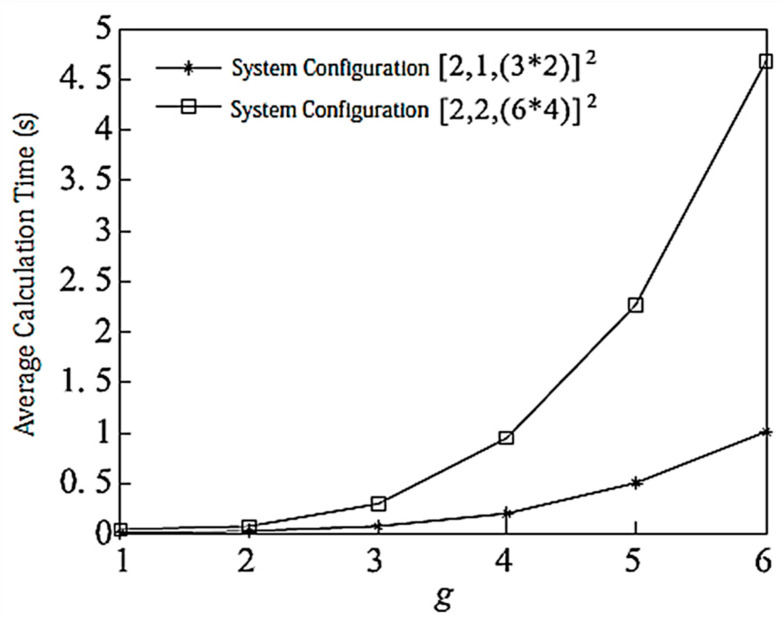
Algorithm complexity with limited feedback bit = 8.

Figure 8 shows the complexity of the proposed algorithm when g is taken as a different value and when the system is configured as ${\left[2,1,\;\left(3\times 2\right)\right]}^{2}$ and ${\left[2,2,\;\left(6\times 4\right)\right]}^{2}$. The simulation is based on 10,000 channel averages, the secondary channel averages, and each user bit = 8. It can be seen from the Figure that the configuration ${\left[2,2,\;\left(6\times 4\right)\right]}^{2}$ has greater computational complexity than ${\left[2,1,\;\left(3\times 2\right)\right]}^{2}$.

### 7.3. Spectral Efficiency Analysis Considering Channel Attenuation with Limited Feedback CSI

**Figure 9:**
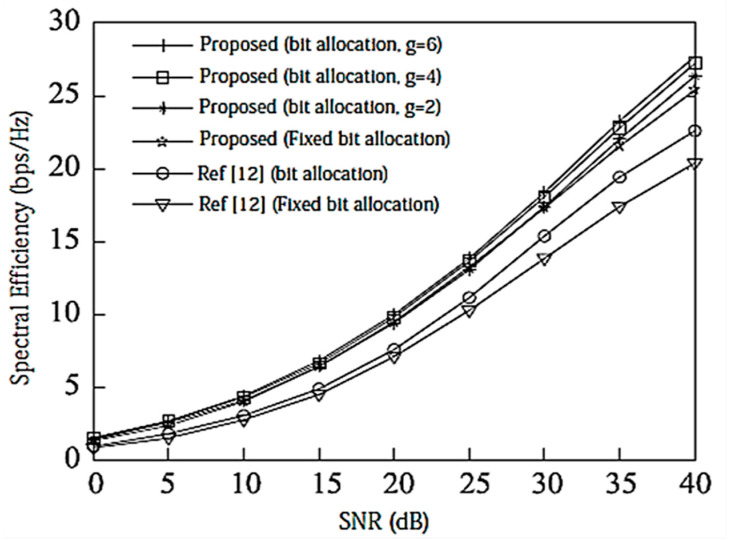
Spectral efficiency analysis of the ${\left[2,1,\;\left(3\times 2\right)\right]}^{2}$ system configuration with a total number of feedback bits $B_{T}=32$.

**Figure 10:**
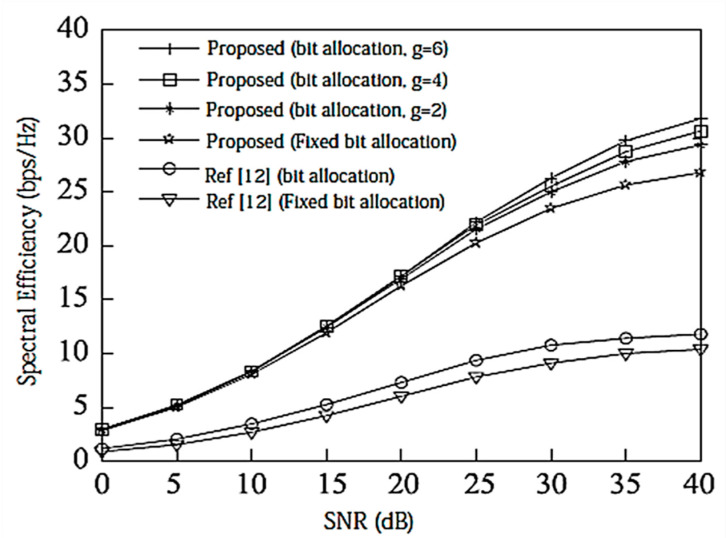
Spectral efficiency analysis of the ${\left[2,2,\;\left(6\times 4\right)\right]}^{2}$ system configuration with a total number of feedback bits $B_{T}=32$.

Assume that the BS radius R=500 m, $d_{0}=200\;m,$ γ=3, and all users fall within the distance from the target BS $D_{s}=700\;m$. The total number of feedback bits for all users is considered here $B_{T}=32$. As can be seen from Figure 9 and Figure 10, the proposed algorithm uses bit allocation and searches for the optimal codeword in the codebook domain, which is close to the ideal precoding system performance. It can also be seen that when the system is configured as ${\left[2,2,\;\left(6\times 4\right)\right]}^{2}$, the performance improvement is better than that of ${\left[2,1,\;\left(3\times 2\right)\right]}^{2}$ which is more obvious from the results.

### 7.4. Average Spectral Efficiency under Ideal CSI

**Figure 11:**
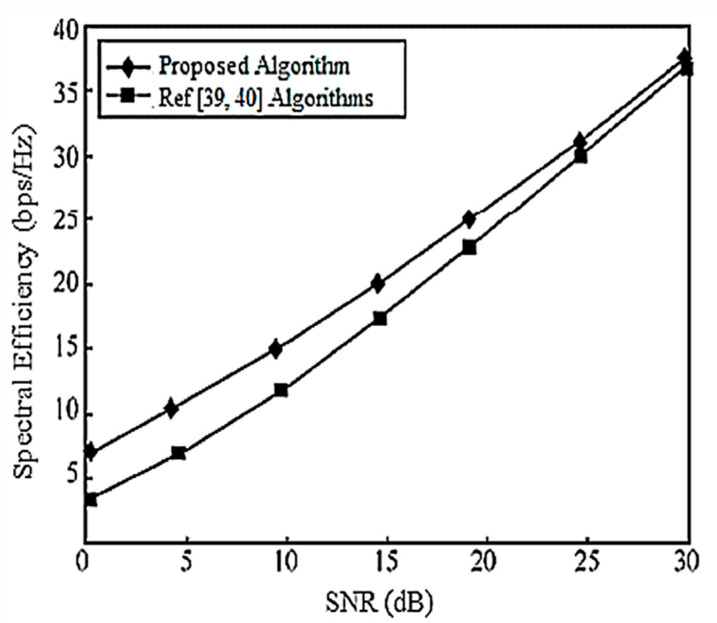
Spectral efficiency analysis of the ${\left[2,1,\;\left(3\times 2\right)\right]}^{2}$ system configuration.

**Figure 12:**
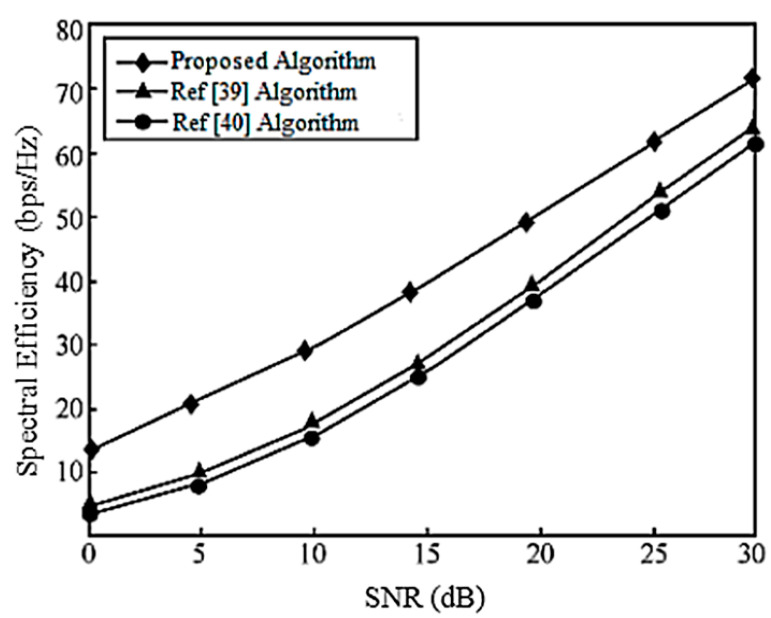
Spectral efficiency analysis of the ${\left[2,2,\;\left(6\times 4\right)\right]}^{2}$ system configuration.

When the system is configured as ${\left[2,1,\;\left(3\times 2\right)\right]}^{2}$ and ${\left[2,2,\;\left(6\times 4\right)\right]}^{2}$, the corresponding system SEs are shown in Figure 11 and Figure 12, respectively, and they are compared with [39,40] for performance evaluation. It is assumed that there is no power loss when the signal propagates to the BS. As can be seen in Figure 11, under the configuration of ${\left[2,1,\;\left(3\times 2\right)\right]}^{2}$, each user has a DoF of 1 (i.e., a single data stream), and [39,40] have the same performance by completely, eliminating the interference algorithm, but the performance is the same. The scheme cannot guarantee the compression of the interference rotation to the optimal receiving direction, and the algorithm in this paper gradually improves the performance by gradually, iteratively rotating and compressing the interference to facilitate the signal receiving direction. 

In the configuration of ${\left[2,2,\;\left(6\times 4\right)\right]}^{2}$, the DoF of each user is 2, and [39] considers the correlation between user data streams when designing the precoding matrix. Its performance is better than that of [40]. The proposed iterative design of the interference suppression matrix gradually rotates the compression interference, and its performance is better than that of both [39,40]. 

### 7.5. Average Spectral Efficiency of Limited Feedback CSI with Equal Loss and High Speeds

**Figure 13:**
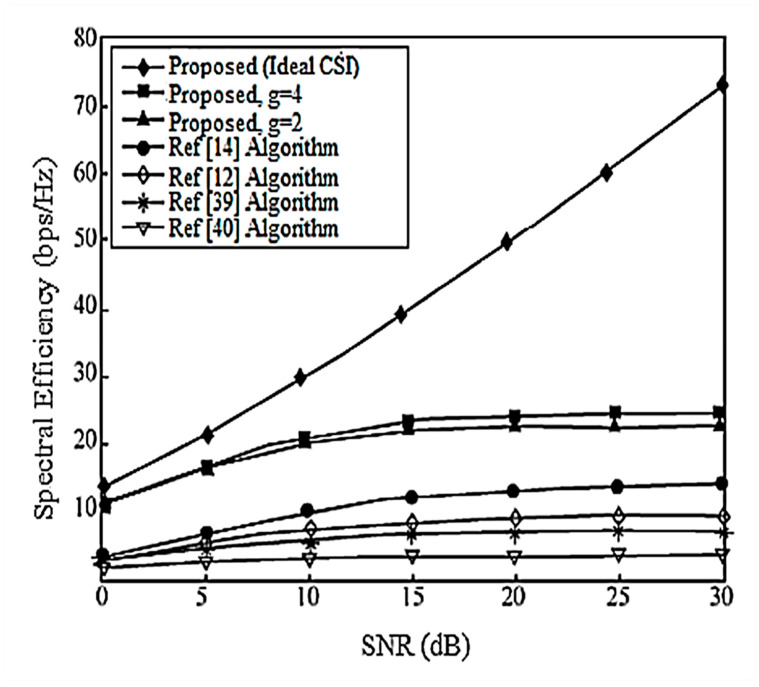
Spectral efficiency analysis of the ${\left[2,1,\;\left(3\times 2\right)\right]}^{2}$ system configuration when bit = 24.

**Figure 14:**
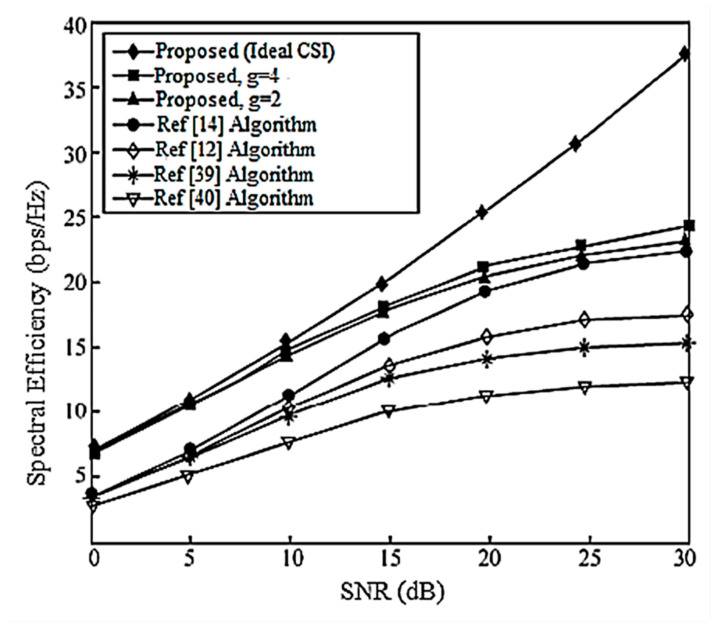
Spectral efficiency analysis of the ${\left[2,2,\;\left(6\times 4\right)\right]}^{2}$ system configuration when bit = 32.

This section provides the average spectral efficiency of the proposed algorithm in the high-speed usage case of transmitter and receiver with limited feedback CSI and equal loss. When the system is configured as ${\left[2,1,\;\left(3\times 2\right)\right]}^{2}$ and ${\left[2,2,\;\left(6\times 4\right)\right]}^{2}$, the corresponding average spectral efficiencies are shown in Figure 13 and Figure 14. Here, it is assumed that there is no power loss when the signal propagates to the BS. It can be seen from these two figures that the proposed algorithm is optimal under the two systems configurations because the optimal precoding matrix designed by the algorithm does not require strict interference alignment so that the interference can remain in the signal space. In order to obtain a larger signal to interference and noise ratio, there is more space for placing interference. Because [40] uses the scheme quantizing the channel matrix, its performance is worse than that of [39] due to its large quantization error. In addition, it can also be seen that:

- In the case of multiple degrees of freedom, the performance of the proposed algorithm is improved relative to the [12,14,39,40].
- The bit allocation scheme of this paper improves the spectrum utilization of the algorithm when the feedback is limited.
- The interference leakage caused by the influence of the quantization error is getting larger and larger, which will limit the spectrum efficiency of the system.

## 8. Conclusions

This paper proposes an efficient Interference Alignment algorithm for maximizing the achievable sum rate and user rate lower-bound of the MIMO-MAC system. In this paper, when the CSI is fed back from the receiver, the performance is deteriorated due to quantization error in the feedback. For this purpose, a direct codeword selection scheme for maximizing the system user rate lower-bound is provided for *K* users in each cell of 2 cells in a MIMO-MAC environment, and a Bit allocation algorithm is used to reduce system and rate loss. The MAX-SINR algorithm is used at the receiver to decode and maximize the lower-bound of the system user rate. From theoretical analysis and simulation results, we can see that as compared with the existing typical algorithms, the proposed algorithm improves the performance of the system to a large extent. A further extension of this research work is to consider more than two cells and consider different channel models for performance evaluations.
